# Acupoint Stimulation for Fibromyalgia: A Systematic Review of Randomized Controlled Trials

**DOI:** 10.1155/2013/362831

**Published:** 2013-12-17

**Authors:** Huijuan Cao, Xun Li, Mei Han, Jianping Liu

**Affiliations:** ^1^Center for Evidence-Based Chinese Medicine, Beijing University of Chinese Medicine, 11 Bei San Huan Dong Lu, Chaoyang District, Beijing 100029, China; ^2^NAFKAM, University of Tromso, Tromso, NO-9037, Norway

## Abstract

*Background*. Acupoint stimulation is popular for treatment of fibromyalgia though there is lack of comprehensive evaluation of current clinical evidence for its effect and safety. *Objective*. To systematically review the beneficial effects and safety of acupoint stimulation for fibromyalgia. *Methods*. We searched six electronic databases for randomized trials on acupoint stimulation for treatment of fibromyalgia. Two authors extracted data and assessed the trial quality independently. RevMan 5.2 software was used for data analyses with effect estimate presented as (standard) mean difference and a 95% confidence interval. We defined minimum, medium, and large SMD effect sizes as 0.3, 0.5, and 0.75. *Results*. 16 RCTs with 1081 participants were involved in this review. Only two trials were evaluated as low risk of bias. Meta-analysis showed that acupuncture alone or combined with cupping therapy was superior to conventional medications on reducing pain scores and/or the number of tender points. However, acupuncture showed no better than sham acupuncture on pain reduction. There was no serious adverse event reported to be related to acupoint stimulation. *Conclusions*. Acupoint stimulation appears to be effective in treating fibromyalgia compared with medications. However, further large, rigorously designed trials are warranted due to insufficient methodological rigor in the included trials.

## 1. Background

As nonspecific rheumatism, fibromyalgia (FM) is a disorder in which typical symptoms are chronic widespread muscular-skeletal pain and stiffness accompanying with fatigue, anxiety, sleep disorder, and/or irritable bowel syndrome [1]. The well known diagnostic criterion for this disease was developed by the American College of Rheumatology (ACR) in 1990 [2], and the latest version of this diagnostic criterion was updated in 2009 [3]. The main purpose of treatment for FM is to alleviate the pain and improve the quality of life for FM patients [4].

Without the curative medications for the entire scope of symptoms and disabilities associated with FM [4], complementary therapies are commonly used by FM patients, such as acupuncture, herbal medicine, and massage. Systematic reviews were also conducted to summarize the clinical evidence of therapeutic effect of those complementary therapies in treating FM. Mayhew and Ernst [5] collected results from five randomized controlled trials in 2007 and demonstrated that due to the small sample size and low methodological quality of included trials, acupuncture could not be recommended for FM. Three years later, other two systematic reviews [6, 7] draw the similar conclusions with three more trials included. Recently, a Cochrane review [8] which is entitled as “Acupuncture for treating fibromyalgia” was published. With nine included trials, it concluded moderate level evidence that acupuncture had no better effect for pain relief compared with sham acupuncture, and there was low to moderate level that evidence showed that acupuncture was better than standard therapy or antidepressant for improving pain. However, all these four reviews only included English articles and mainly observed the comparison between acupuncture and sham acupuncture. In our previous review [9], 12 trials focusing on acupuncture were included, and the result showed that even though acupuncture showed no significant effect compared with sham acupuncture (MD −0.55, 95%CI −1.35 to 0.24, *P* = 0.17, *I* ^2^ = 69%) on pain reduction, there were significant effect of acupuncture on reducing the number of tender points (MD −3.21, 95%CI −4.23 to −2.11, *P* < 0.00001, *I* ^2^ = 0%), and pain scores (MD −1.78, 95%CI −2.24 to −1.32, *P* < 0.00001, *I* ^2^ = 0%) compared with conventional medications. We also found that, besides acupuncture, other point stimulation therapies, such as cupping therapy, were commonly used in clinics for treatment of FM.

Acupoint stimulation therapy includes acupuncture, cupping therapy (which involves applying suction by placing a vacuumized, usually by fire, cup or jar on points or affected body surfaces to induce local hyperemia or haemostasis), moxibustion (which involves the controlled burning of material, typically mugwort herb, at certain points or areas of the body surface), point injection (which involves injecting medication into an acupuncture point), point embedding (which involves embedding in the skin over the point with a small needle (s) or medicated catgut), or combination of two or more of those acupoint stimulation.

In traditional Chinese medicine (TCM) theory, stagnation of *qi *activity leads to the stasis of blood which causes pain [10]. Therefore, the potential mechanism of acupoint stimulation for FM is to regulate the *qi* and blood, combined with dispelling cold and removing damp. Though acupoint stimulation was popularly employed in treating FM, there is no systematic review that evaluates the clinical evidence of all types of acupoint stimulation. This systematic review aims to update the evidence from RCTs to evaluate the therapeutic effect and safety of different types of acupoint stimulation therapies for FM.

## 2. Methods

### 2.1. Inclusion Criteria

We included parallel-group RCTs of any kind of point stimulation therapies including acupuncture, cupping therapy, point injection, point catgut embedding, or moxibustion, compared with no treatment, placebo, or conventional medication in patients with FM. We also included combined therapy with acupoint stimulation and other interventions compared with other interventions in RCTs, or combined therapy of two kinds of point stimulation therapies compared with medication or other interventions. FM should be diagnosed according to recognized criteria. Primary outcome was change of pain intensity, and secondary outcomes included improvement of relevant symptoms, such as depression or quality of life and adverse events. There was no limitation on language and publication type.

### 2.2. Identification and Selection of Studies

We searched China Network Knowledge Infrastructure (CNKI) (1979–2013), Chinese Scientific Journal Database VIP (1989–2013), Wan Fang Database (1985–2013), Chinese Biomedicine (Sino-Med) database (1978–2013), PubMed (1966–2013), and the Cochrane Library (Issue 5, 2013), and all the searches ended at May 2013. The search terms included “fibromyalgia,” “fibrosis,” “fibrositis,” “myofascitis,” or “myofibrositis” combined with “acupuncture,” “electroacupuncture,” “auricular therapy,” “acupoint,” “point embedding,” “point injenction,” “cupping,” “moxibustion,” or “meridian.” Two authors (Huijuan Cao and Mei Han) selected studies for eligibility and checked against the inclusion criteria independently.

### 2.3. Data Extraction and Quality Assessment

Two authors (Huijuan Cao and Mei Han) extracted the data from the included trials independently. Selection bias (random sequence generation and allocation concealment), performance bias (blinding of participants and personnel), detection bias (blinding of outcome assessment), attrition bias (incomplete outcome data), reporting bias (selective reporting), and other bias were assessed according to the criteria from the *Cochrane Handbook for Systematic Reviews of Intervention* [11]. There were three potential bias judgments: low risk, high risk, and unclear risk. A judgment of low risk was made when all the seven items met the criteria as “low risk,” a judgment of high risk of bias was made when at least one of the seven items was assessed as “high risk.”

### 2.4. Data Analysis

Data were summarized using risk ratio (RR) with 95% confidence intervals (CI) for binary outcomes or mean difference (MD)/standard mean difference (SMD) with 95%CI for continuous outcomes. For pain reduction, at least 30% difference of VAS scores are needed to be detected after treatment to achieve the minimum clinical therapeutic effect [12]. Thus, we defined minimum, medium, and large SMD effect sizes as 0.3, 0.5, and 0.75. We used Revman5.2 software from the Cochrane Collaboration for data analyses. Meta-analysis was used if the trials had an acceptable homogeneity on study design, participants, interventions, control, and outcome measures. Statistical heterogeneity was tested by examining *I* ^2^ [13], meaning that an *I* larger than 50% indicates the possibility of statistical heterogeneity. Both fixed effect model and random effects model were used if there was possibility of statistical heterogeneity among trials. If *I* ^2^ was less than 50%, only a fixed effect model was used for meta-analysis. Publication bias was explored by funnel plot analysis. Subgroup analyses were conducted to determine the evidence for the different point choice or the different treatment duration if data were sufficient. Sensitivity analyses were in order to determine whether the conclusions were differed if (1) eligibility was restricted to studies without high risk of bias; (2) a fixed effect/random effect model had been applied.

## 3. Results

### 3.1. Description of Studies

**Figure 1:**
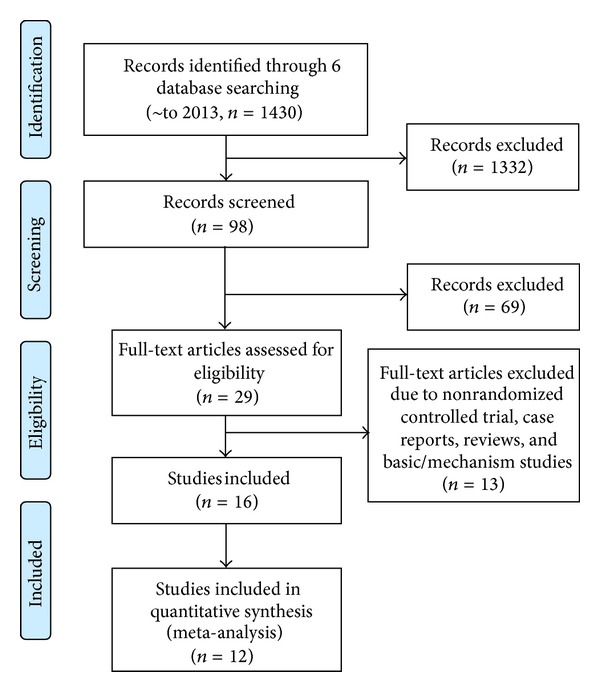
Flow chart.

After primary searches in six databases, 1430 citations were identified, as the majority was excluded due to obvious ineligibility, and full text papers of 29 studies were finally retrieved. Finally, 16 randomized trials [14–29] were included in this review (Figure 1), and one trial was published as two separate papers [21, 30] and two unpublished dissertations [31, 32] two trials were reported as dissertations [24, 29], one trial [26] was reported in a conference, nine [14, 16, 18–20, 22, 25, 27, 28] of the remaining 13 trials were published in English scientific journals, and other four trials were published in Chinese journals. The characteristics of included trials were listed in Table 1 (Characteristics of included studies).

The 16 RCTs involved a total of 1081 patients with FM (an average of 30 patients per group). The participants were aged from 17 to 73 years, and the disease duration was from 4 months to 6 years. All except one trial used ACR 1990 as the diagnostic criteria, and the remaining one trial [22] applied the International Academy of Soreness Research (IASR) [33] for diagnosing FM. The interventions included acupuncture (electroacupuncture, auricular acupuncture), cupping, moxibustion and combinations of acupuncture, and cupping. The controls included no treatment, sham acupuncture or medications. The treatment duration ranged from 2 to 13 weeks. Change of visual analogue scores (VAS) as the major outcome measurement was reported in 11 trials [14–18, 22–24, 27, 29]. Eight trials [15, 18, 21, 23, 24, 27–29] calculated the change of number of tender points, and six trials [18–21, 26, 27] reported results of the McGill Pain Questionnaire (MPQ), Present Pain Intensity (PPI), or Fibromyalgia Impact Questionnaire (FIQ) for assessing intensity of pain. Three trials [15, 17, 21] used the Hamilton Depression Scale (HAMD) to assess depression. Four trials [14, 16, 17, 19] evaluated quality of life or quality of sleep. Six trials [15, 17, 21, 24, 26, 29] applied four categories to evaluate treatment effects including cure (symptoms disappeared and no tender points exist), markedly effective (symptoms improved more than 50%), effective/improve (symptoms improved between 25% and 50%), and ineffective (symptoms improved less than 25%).

### 3.2. Methodological Quality

**Figure 2:**
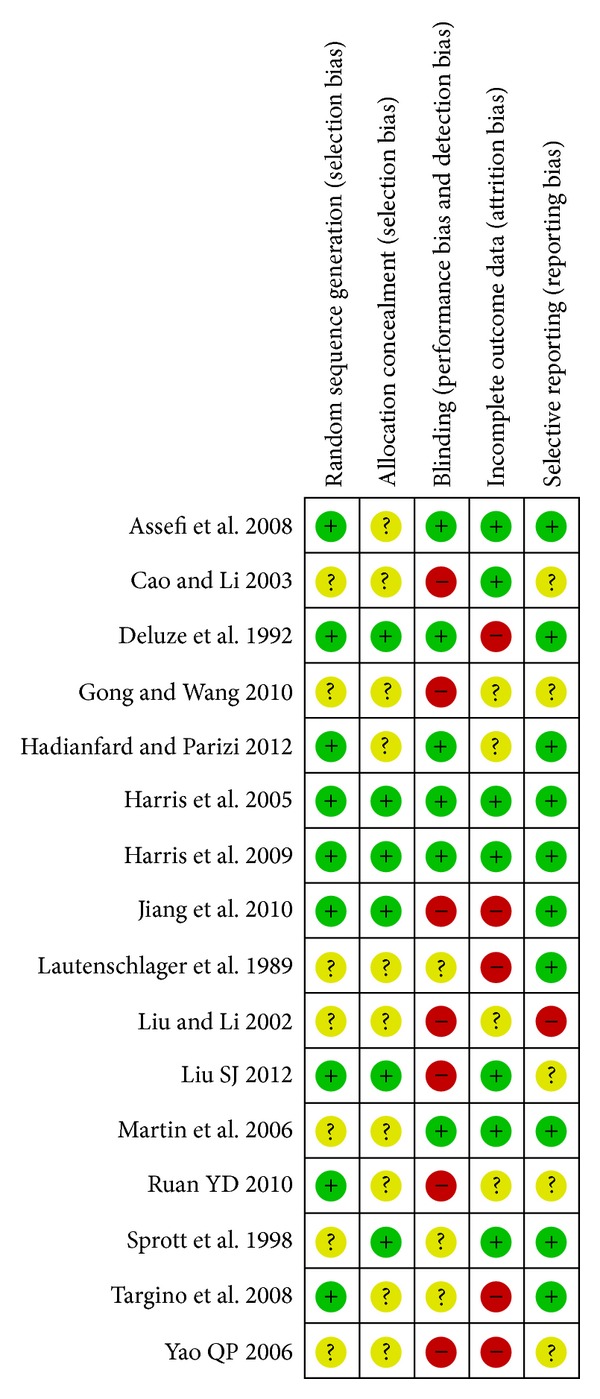
Methodological quality summary: review authors' judgments about each methodological quality item for each included study.

According to our predefined quality assessment criteria, two [19, 20] out of the 16 trials (12.5%) were evaluated as low risk of bias, ten trials were evaluated as high risk of bias, while the other four included trials [14, 18, 27, 29] as unclear risk of bias (Figure 2 Methodological quality summary: review authors' judgments about each methodological quality item for each included study). The sample size varied from 20 to 186 participants, with average of 30 patients per group. Only three trials [16, 18, 21] reported prior sample size calculation, nine trials [14, 16, 18–21, 24, 28] described randomization procedures (using random number table or computer generation of random numbers), and six trials [16, 19–21, 24, 27] reported adequate allocation concealment. Three trials [16, 19, 20] blinded both patients and outcome assessors, four trials [14, 18, 21, 25] blinded only the outcome assessors. Five trials [16, 21, 22, 28, 29] reported the number of dropouts, and none of these trials used intention-to-treat analysis.

### 3.3. Effect Estimates

Thirteen trials [14, 16–20, 25, 27, 29] tested acupuncture for treating FM. Eight trials [14, 16, 19, 20, 22, 25, 27, 28] compared acupuncture or electroacupuncture with sham acupuncture or sham electroacupuncture, and five trials [17, 18, 23, 24, 29] compared acupuncture with medications.

One trial [26] observed the comparison between moxibustion and medication (amitriptyline 10–30 mg daily) for treating FM.

Three trials [15, 21, 24] tested therapeutic effect of combination therapies of acupoint stimulation for fibromyalgia. Two trials [15, 21] compared acupuncture and cupping therapy plus medication with medication only (amitriptyline 25 mg daily), one trial [21] compared acupuncture and cupping therapy with medications, and one trial [24] compared acupuncture plus point injection with amitriptyline (25–50 mg daily).

Due to the insufficient number of the included trials in one meta-analysis, we could not perform a meaningful funnel plot analysis. Results of meta-analysis were listed in Table 2 (estimate effect of included trials in meta-analyses), while results of each individual trials which could not be synthesized in a meta-analyses were concluded in Table 3 (characteristics of randomized controlled trial outside of meta-analysis).

### 3.4. Therapeutic Effect of Acupoint Stimulation for Pain Relieving

Neither subtotal meta-analysis nor total meta-analysis showed any difference between acupuncture and sham acupuncture on reducing pain (changes between baseline and post-treatment: SMD −0.09, 95%CI −0.32 to 0.14, *I* ^2^ = 2%, random model, *P* = 0.44, 6 trials; at posttreatment: SMD −0.22, 95%CI −0.51 to 0.07, *I* ^2^ = 26%, random model, *P* = 0.13, 6 trials). However, one subtotal meta-analysis showed that electroacupuncture was superior to sham electroacupuncture regarding pain reduction after treatment (SMD −0.42, 95%CI −0.77 to −0.06, *I* ^2^ = 0%, random model, *P* = 0.02, 3 trials). Two trials [22, 27], which could not be included in meta-analysis, also showed no difference between acupuncture and sham acupuncture or no treatment on pain relieve (*P* > 0.05), respectively. The main findings of these trials were presented in Table 3 (characteristics of RCTs outside of meta-analysis). Meta-analysis of five trials [17, 18, 23, 24, 29] showed that acupuncture was better than antidepression drugs (amitriptyline 25 mg daily, subtotal: SMD −0.60, 95%CI −0.93 to −0.27, *I* ^2^ = 22%, random model, *P* = 0.0004, 4 trials) or the analgesic antipyretic (ibuprofen 0.9 g daily) with regard to pain reduction according to VAS scores (total: SMD −0.74, 95%CI −1.13 to −0.35, *I* ^2^ = 55%, random model, *P* = 0.0002, 5 trials) and the tender points (MD −2.38, 95%CI −3.40 to −1.37, *I* ^2^ = 0%, fixed model, *P* < 0.00001, 3 trials).

Two trials [15, 21] showed that a combination of acupuncture and cupping therapy plus medications was significantly better than medications (amitriptyline 25 mg daily) alone regarding pain reduction (SMD −1.65, 95%CI −2.10 to −1.31, *I* ^2^ = 0%, fixed model, *P* < 0.00001, 2 trials). However, one trial [21] showed no difference between acupuncture plus cupping therapy and medications (amitriptyline 25 mg daily) for this outcome.

Moxibustion (SMD −1.46, 95%CI −2.00 to −0.91, *P* < 0.00001, 1 trials) or combination of acupuncture and point injection (SMD −1.53, 95%CI −2.09 to −1.96, *P* < 0.00001, 1 trials) was superior to amitriptyline (10–50 mg daily) regarding pain reduction.

### 3.5. Therapeutic Effect of Acupoint Stimulation for Improving Depression

No difference between electroacupuncture and sham electroacupuncture was found for improving depression (SMD −0.33, 95%CI −0.90 to 0.23, *P* = 0.25, 1 trial), which was also true for the combination of acupuncture and cupping therapy (MD 0.90, 95%CI −0.68 to 2.48, *P* = 0.26, 1 trial).

Meta-analysis showed that acupuncture was better than antidepression drugs (amitriptyline or fluoxetine) for improving depression (SMD −0.67, 95%CI −1.10 to −0.25, *I* ^2^ = 0%, fixed model, *P* = 0.02, 2 trials). Two trials also demonstrated that combination of acupuncture and cupping therapy plus medications was better than medications alone for treating FM related depression (*P* < 0.01); however, meta-analysis could not be conducted due to the significant statistical heterogeneity between trials.

### 3.6. Therapeutic Effect of Acupoint Stimulation for Improving Sleep Quality

Three trials [17, 24, 29] evaluated therapeutic effect of acupuncture for sleep quality after treatment that meta-analysis showed acupuncture was superior to amitriptyline (25–50 mg daily) for improving sleep quality (SMD −0.32, 95%CI −0.63 to −0.01, *I* ^2^ = 0%, fixed model, *P* = 0.04, 3 trials).

One trial [24] showed a significant advantage of combination of acupuncture and point injection on improving sleep quality compared with amitriptyline (25–50 mg daily, SMD −0.94, 95%CI −1.46 to −0.42, *P* = 0.0004, 1 trial).

### 3.7. Therapeutic Effect of Acupoint Stimulation for Improving FM Related Fatigue

No superior effect of acupuncture was found for fatigue relieve, neither compared to sham acupuncture (SMD −0.05, 95%CI −0.41 to 0.30, *I* ^2^ = 0%, fixed model, *P* = 0.77, 3 trials) nor compared with anti-depression drugs (fluoxetine 20 mg daily, SMD −0.27, 95%CI −0.99 to 0.45, *P* = 0.46, 1 trial).

### 3.8. Adverse Events

Only three trials described adverse events [14, 21, 29], all of which were related to acupuncture or medications. The adverse events of acupuncture included bruising, nausea (3%), fainting (0.3–5%), discomfort at the sites of needle insertion or simulated needle insertion (37%), and a small amount of bleeding (0.02%). One trial [14] reported that patients assigned to simulated acupuncture (29%) had significantly less discomfort than those assigned to real acupuncture (61%), acupuncture for an unrelated condition (70%), or sham needling (64%). Few patients (up to 8.3%) with palpitations, fainting, dry mouth, fatigue, and constipation were reported from control medications.

The remaining thirteen trials did not report related information about adverse events during the treatment.

No serious adverse event was reported in all included trials.

## 4. Discussions

Our review with 16 included trials demonstrates that acupuncture or combination of acupuncture and cupping therapy was significantly more effective than conventional medications (anti-depression drugs or analgesic antipyretic) on reducing pain and improving the FM related symptoms (such as depression or fatigue), and however, the therapeutic effects of other types of acupoint stimulation are uncertain due to the limited numbers of clinical trials.

There are several limitations of this review. The quality of the included studies is generally poor, which indicates high or unclear risk of bias due to insufficient reporting of methodological components from the trials. There was unclear description of randomization procedure and lack of blinding in half of the trials which may create potential performance bias and detection bias as patients and researchers might be aware of the therapeutic interventions. Intention-to-treat analysis was not applied in most of the included trials. We were limited in our ability to perform meta-analysis due to the incomplete outcome data reporting in three included trials [14, 25, 28]. Outcomes such as *cure, markedly effective, effective, or ineffective* were used in six trials, these are not validated, and the finding will be hard to interpret. Consequently, the interpretation of these positive findings needs to be cautious, and the study methodology needs to be improved for future confirmatory studies.

Though we could not perform a meaningful funnel plot due to the insufficient number of included trials in meta-analysis, there was potential publication bias among included trials. All the nine English publications reported no significant statistical difference between groups, but five out of seven Chinese publications showed significant advantages of acupoint stimulation compared with control. One of the potential reasons is all but one trial [18] conducted outside China employed the comparison between acupuncture and sham acupuncture, but sham acupuncture may not be appropriate as a placebo against which to evaluate the therapeutic effect of real acupuncture [34]. Due to the sociocultural background, Chinese participants may have a preference for acupuncture treatment compared to a pharmacological intervention, which may create potential performance bias and affect the results of assessment with patients report outcome. Besides, no trial used syndrome differentiation for acupuncture point selection.

Comparing to previous systematic reviews [6–8] which assessed acupuncture, our review included two more English publications and four more trials conducted in China. Results of the comparison between acupuncture and sham acupuncture were similar to other reviews due to the same data source; however, regardless of the poor methodological quality of included trials, we found that acupuncture compared to medications showed significant therapeutic effects on improving FM related symptoms (including pain, fatigue, depression, or insomnia). We also assessed trials focusing on the effect of other acupoint stimulation, but no confirmed conclusions could be drawn due to the insufficient number of relevant trials.

Considering the small sample sizes and unclear or high risk of bias of the current included trials, we suggest that trials with rigorous designing should be conducted to further confirm the effectiveness of acupoint stimulation in treating FM. Although there is not ideal placebo for acupoint stimulation and blinding of the patients and practitioners might be very difficult for acupuncture or herbal medicine, blinding of outcome assessors should be attempted as far as possible to minimize performance and assessment biases. Choosing outcome measures should be based on international consensus and include continuous data and daily average pain scores from baseline to study completion. Using appropriate methods (such as intention to treat analysis) to deal with missing data is vital as is the application of well-defined diagnostic criteria, such as ACR 2009. Reporting of trials should follow the Consolidated Standards of Reporting Trials (CONSORT) [35] or Standards for Reporting Interventions in Clinical Trials of Acupuncture (STRICTA) [36] to explicitly explain the processes involved transparently. Our preliminary conclusions suggest that patients with FM might benefit from acupoint stimulation, especially acupuncture or combination of acupuncture and cupping therapy. However, more high quality clinical evidence is needed to further testify this conclusion.

### 4.1. Key Message

There is low level evidence showing that acupuncture or a combination of acupuncture and cupping therapy was significantly more effective than conventional medications (antidepression drugs or analgesic antipyretic) on reducing pain and improving the FM related symptoms (such as depression or fatigue).

Acupuncture seems to have no better effect than sham acupuncture with regard to pain relieve in patients with fibromyalgia according to the current moderate level clinical evidence.

The therapeutic effects of other types of acupoint stimulation are uncertain due to limited numbers of clinical trials.

## Figures and Tables

**Table 1:** Characteristics of included studies.

Study ID	Diagnostic criteria	Sample size (T/C, male/female)		Age (yr, T/C)		Duration of disease (month, T/C)		Experimental intervention	Control intervention	Duration of treatment	Outcomes
Assefi et al. 2005 [14]	ACR 1990	3/22	2/69	Unclear		144 ± 216	112.08 ± 120.48	Acupuncture on points chose to treat fibromyalgia according to TCM theory for 30 min, twice weekly	One of three sham acupuncture (false points, not insertion, or unrelated points) for 30 min, twice weekly	12 weeks	Visual Analogue Scores (VAS) for pain, fatigue, Sleep, and overall well-being; SF-36 for physical and mental function; adverse effects

Cao and Li 2003 [15]	ACR 1990	28	28	42.1 ± 14.5		19.3 ± 15.1		Acupuncture plus moving cupping therapy on bilateral Jiaji points, once every 3 days, plus seroxat 20 mg daily	Seroxat 20 mg daily	4 weeks	Hamilton Depression Scale (HAMD); VAS; number of tender points; effective rate

Deluze et al. 1992 [16]	ACR 1990	3/33	13/21	46.8 ± 2.3	49 ± 2	172.8 ± 40.8	82.8 ± 15.6	Electroacupuncture on 4–10 common points with electrostimulation 1–99 Hz, 10 mA, twice weekly for 6 sessions	Sham electroacupuncture on false points (20 mm away from the point which has been chosen for real electroacupuncture) twice weekly for 6 sessions	3 weeks	Pain threshold; number of analgesic tablets; regional pain score; VAS for pain; Sleep Quaty; morning stiffness; patients and evaluation physicians appreciation of the patients general status

Gong and Wang 2010 [17]	ACR 1990	9/21	11/19	35 ± 8	34 ± 6	15.0 ± 3.5	13.0 ± 2.5	Acupuncture at Ashi points and lower Dantian (CV4 and CV6) for 30 min, once daily to twice weekly	Amitriptyline 25 mg twice daily added to 150–300 mg daily for 2 months, then 50–150 mg per month for another month	12 weeks	VAS for pain; sleep quality; HAMD; effective rate

Hadianfard and Parizi 2012 [18]	ACR 1990	0/15	0/15	43.86 ± 7.9	44.2 ± 10.8	82.8 ± 68.4	79.6 ± 69.8	Acupuncture on ST36, GB34, RN6, SP6, LI4, ST44, BL40, HT7, and DU20 for 30 min, three sessions weekly	Fluoxetine 20 mg every morning	8 weeks	VAS; number of tender points; Fibromyalgia impact questionnaire (FIQ)

Harris et al. 2005 [19]	ACR 1990	0/29 3/27	4/24 1/26	46 ± 10.1 44.5 ± 10.9	51.3 ± 10.0 48.1 ± 10.9	66 ± 44.52 63.12 ± 57.96	62.04 ± 50.88 69.24 ± 49.2	Acupuncture at DU20, LI11, LI4, GB34, bilateral ST36, SP6, Liv 3, and ear-shenmen with manual stimulation for 20 min once to three times weekly Acupuncture at above points without manual stimulation for 20 min once to three times weekly	Sham acupuncture on nontraditional site with manual stimulation for 20 min, once to three times weekly Sham acupuncture at above site without manual stimulation for 20 min, once to three times weekly	13 weeks	Numeric Rating Scale (NRS), Multi-Dimensional Fatigue Inventory questionnaire, Reliability of Change Index (RC), SF-36 questionnaire for function

Harris et al. 2009 [20]	ACR 1990	0/10	0/10	44.3 ± 13.6		unclear		Acupuncture at DU20, LI11, LI4, GB34, SP6, Liv 3, ear-shenmen, and bilateral ST36 with manual stimulation	Sham acupuncture without penetration on nontraditional site for 20 min	Unclear	Short form McGill pain questionnaire (MPQ); u-opioid receptors (MORs)

Jiang et al. 2010 [21]	ACR 1990	19/43 15/49	9/51	41.9 ± 9.85 43.89 ± 10.53	42.83 ± 11.27	22.82 ± 12.26 21.64 ± 13.80	21.3 ± 12.84	Electroacupuncture at B42, B44, B47, B49, and B52 with 2 Hz/50 Hz stimulation for 20 min, then moving cupping along bilateral 1.6, 4.8, and 10 cm beside the spine for 5 min, three times weekly Electroacupuncture and moving cupping, plus amitriptyline 25 mg once every night	Amitriptyline 25 mg once every night	4 weeks	MPQ; HAMD; respond time; adverse events; laboratory tests; effective rate; number of tender points

Lautenschlager et al. 1989 [22]	Clinical symptoms	25	25	Unclear		unclear		Acupuncture at 8–10 of 25 predefined points with manual stimulation for 45 min, 6 sessions	Sham acupuncture (nonpenetrating) with disconnected laser equipment	2 weeks	VAS

Liu and Li 2002 [23]	IASR	4/26	3/27	29–68	31–69	45.6 ± 16.8	46.8 ± 14.4	Acupuncture at Ashi points with heavy manual stimulation once every 2 min for 6 min, once daily	Ibuprofen 0.3 g three times daily	2 weeks	VAS, number of tender points

Liu 2012 [24]	ACR 1990	5/27 4/28	4/28	39 ± 6.7 41 ± 6.4	40 ± 6.6	23 ± 8.7 23 ± 8.5	23 ± 8.4	Acupuncture at GV20, BI18, BI20, Jiaji, P6, and Ashi for 30 min, once daily Acupuncture at above points, then injection with 2 mL Vit B12 at BI23 and ST36 once daily	Amitriptyline 25–50 mg once per night	4 weeks	VAS, number of tender points, Ahtens for insomnia assessment; adverse events; effective rate

Martin et al. 2006 [25]	ACR 1990	25	25	51.7 ± 14.1		47.9 ± 11.2	unclear	Acupuncture at bilateral LI4, ST36, Liv2, SP6, pericardium 6, heart 7, and axial paramedian points along the bladder meridian at cervical spine during first 3 sessions and at the lumbar spine during last 3 sessions; electrical stimulation applied at 2 Hz between LI4 and ST36, and 10 Hz over axial circuits for 20 min once every 2 to 4 days	Sham electroacupuncture (not insertion) 20 min once every 2 to 4 days	2 to 3 weeks	FIQ, Multidimensional Pain Inventory (MPI)

Ruan 2010 [26]	ACR 1990	6/27	5/28	49.0 ± 3.6	50 ± 2.1	9 ± 3.8	10 ± 3.2	Moxibustion at two of the below points: Ashi, Jiaji, GV4, DU3, CV9, GV14, LI10, GB34, B40, B12, once daily	Amitriptyline 10–30 mg twice daily	4 weeks	MPQ; effective rate

Sprott 1998 [27]	ACR 1990	10	10 10	55		unclear		Electroacupuncture on points according to TCM twice weekly plus basic therapy	Sham acupuncture with disconnected laser equipment plus basic therapy Sham acupuncture with nonpuncture treatment plus basic therapy	2–4 weeks	Number of tender points; VAS

Targino 2008 [28]	ACR 1990	0/34	0/24	52.09 ± 10.97	51.17 ± 11.20	118.8 ± 117.3	93 ± 75.25	Acupuncture at Ex-HN-3 and bilateral LR3, LI4, PC6, GB34, SP6 for 20 min twice weekly plus standard care (same with control)	Tricyclic antidepressants 12.5–75 mg once daily; walk for 30 min twice weekly; mental relaxation exercise for another 30 min; stretching excise twice weekly	10 weeks	VAS; number of tender points; mean pressure pain threshold (PPT); SF-36

Yao 2006 [29]	ACR 1990	5/15	4/15	40 ± 8.6	41 ± 8.5	12 ± 3.4	12 ± 4.5	Acupuncture at G20, SI11, LI11, P6, LI4, BI18, Liv14, ST36, SP6, SP10, GB34, SP9, and Ashi for 30 min, once daily	Amitriptyline 30–50 mg once per night	4 weeks	VAS, number of tender points, Ahtens for insomnia assessment; adverse events; effective rate

T: treatment, c: control.

ACR: American College of Rheumatology criteria for the classification of fibromyalgia.

IASR: International Academy of Soreness Research.

**Table 2:** Estimated effect sizes of included trials in meta-analyses.

Trials	Interventions	Estimate effects	*P* value	*I* ^2^
(1) Changes of VAS scores for pain				
(1.1) Therapeutic effect of acupuncture				
(1.1.1) Acupuncture versus sham acupuncture				
Assefi et al. 1989 [14]	Acupuncture versus sham acupuncture on false acupoints	0.23 [−0.23, 0.68]		
Harris et al. 2005 [19]	Acupuncture on traditional site versus acupuncture on nontraditional site	0.28 [−0.33, 0.89]		
Harris et al. 2005 [19]	Acupuncture on traditional site with stimulation versus acupuncture on nontraditional site with stimulation	−0.30 [−0.98, 0.38]		
Harris et al. 2009 [20]	Acupuncture versus sham acupuncture without penetration on nontraditional site	−0.14 [−1.02, 0.74]		
Lautenschlager et al. 1989 [22]	Acupuncture versus sham acupuncture with disconnected laser equipment	−0.55 [−1.21, 0.11]		
Subtotal (random model)		SMD 0.04 [−0.37, 0.28]	0.79	24%
(1.1.2) Electroacupuncture versus sham electroacupuncture				
Lautenschlager 1989 [22]	Electroacupuncture versus sham electroacupuncture on false acupoints	−0.30 [−0.84, 0.23]		
Martin et al. 2006 [25]	Electroacupuncture versus sham electroacupuncture without insertion	−0.12 [−0.68, 0.44]		
Subtotal (random model)		SMD −0.22 [−0.60, 0.17]	0.27	0%
Overall (random model)		**SMD −0.09** [**−0.32, 0.14**]	**0.44**	**2%**

(2) VAS scores for pain after treatment				
(2.1) Therapeutic effect of acupuncture				
(2.1.1) Acupuncture versus sham acupuncture				
(2.1.1.1) Acupuncture versus sham acupuncture				
Assefi et al. 1989 [14]	Acupuncture versus sham acupuncture on false acupoints	0.24 [−0.37, 0.84]		
Harris et al. 2005 [19]	Acupuncture on traditional site versus acupuncture on nontraditional site	0.31 [−0.30, 0.92]		
Harris et al. 2005 [19]	Acupuncture on traditional site with stimulation versus acupuncture on nontraditional site with stimulation	−0.46 [−1.15, 0.22]		
Harris et al. 2009 [20]	Acupuncture versus sham acupuncture without penetration on nontraditional site	−0.65 [−1.55, 0.26]		
Subgroup (random model)		SMD 0.07 [−0.53, 0.38]	0.75	43%
(2.1.1.2) Electroacupuncture versus sham electroacupuncture				
Lautenschlager et al. 1989 [22]	Electroacupuncture versus sham electroacupuncture on false acupoints	−0.56 [−1.10, −0.02]		
Martin et al. 2006 [25]	Electroacupuncture versus sham electroacupuncture without insertion	−0.28 [−0.84, 0.28]		
Sprott 1998 [27]	Electroacupuncture plus basic therapy versus sham electroacupuncture with nonpuncture treatment plus basic therapy	−0.38 [−1.27, 0.50]		
Subgroup (random model)		SMD −0.42 [−0.77, −0.06]	0.02	0%
Overall (random model**)**		**SMD −0.22** [**−0.51, 0.07**]	**0.13**	**26%**
(2.1.2) Acupuncture versus medications				
(2.1.2.1) Acupuncture versus anti-depression drugs				
Gong and Wang 2010 [17]	Acupuncture versus amitriptyline	−0.98 [−1.52, −0.44]		
Hadianfard and Parizi 2012 [18]	Acupuncture versus fluoxetine	−0.40 [−1.12, 0.33]		
Liu 2012 [24]	Acupuncture versus amitriptyline	−0.66 [−1.16, 0.16]		
Yao 2006 [29]	Acupuncture versus amitriptyline	−0.20 [−0.82, 0.43]		
Subtotal (random model)		SMD −0.60 [−0.93, −0.27]	0.0004	22%
(2.1.2.2) acupuncture versus analgesic-antipyretic				
Liu and Li 2002 [23]	Acupuncture versus ibuprofen	−1.34 [−1.90, −0.77]	<0.00001	NA
Overall (random model)		**SMD −0.74** [**−1.13, −0.35**]	**0.0002**	**55%**
(2.2) Therapeutic effect of combination of acupuncture and cupping therapy				
(2.2.1) Combination of acupuncture and cupping therapy plus medications versus medications alone				
Jiang et al. 2010 [21]	Acupuncture plus cupping therapy and seroxat versus seroxat alone	−1.63 [−2.18, −1.08]		
Jiang et al. 2010 [21]	Acupuncture plus cupping therapy and amitriptyline versus amitriptyline	−1.77 [−2.74, −0.80]		
Overall (fixed model)		**SMD −1.65** [**−2.10, −1.31**]	**<0.00001**	**0%**
(2.2.2) Combination of acupuncture and cupping therapy versus medications				
Jiang et al. 2010 [21]	Acupuncture plus cupping therapy and amitriptyline versus amitriptyline	SMD −0.21 [−0.57, 0.15]	0.25	NA
(2.3) therapeutic effect of moxibustion				
Ruan 2010 [26]	Moxibustion versus amitriptyline	SMD −1.46 [−2.00, −0.91]	<0.00001	NA
(2.4) Therapeutic effect of combination of acupuncture and point injection				
Liu 2012 [24]	Acupuncture combined with point injection (Vit B12) versus amitriptyline	SMD −1.53 [−2.09, −1.96]	<0.00001	NA

(3) No. of tender points after treatment				
(3.1) Therapeutic effect of acupuncture				
(3.1.1) Acupuncture versus medications				
(3.1.1.1) Acupuncture versus anti-depression drugs				
Liu 2012 [24]	Acupuncture versus amitriptyline	−1.50 [−3.46, 0.46]		
Yao 2006 [29]	Acupuncture versus amitriptyline	−1.70 [−4.22, 0.82]		
Subtotal (fixed model)		MD −1.58 [−3.12, −0.03]	0.05	0%
(3.1.1.2) acupuncture versus analgesic-antipyretic				
Liu and Li 2002 [23]	Acupuncture versus ibuprofen	MD −3.00 [−4.35, −1.65]	<0.0001	NA
Overall (fixed model)		**MD −2.38** [**−3.40, −1.37**]	**<0.00001**	**0%**
(3.2) Therapeutic effect of combination of acupuncture and cupping therapy				
(3.2.1) Combination of acupuncture and cupping therapy versus western medications				
Jiang et al. 2010 [21]	Acupuncture plus cupping therapy and amitriptyline versus amitriptyline	MD −0.84 [−1.72, 0.04]	0.06	NA
(3.2.2) combination of acupuncture and cupping therapy plus western medications versus medications alone				
Jiang et al. 2010 [21]	Acupuncture plus cupping therapy and seroxat versus seroxat alone	−3.90 [−6.29, −1.51]		
Jiang et al. 2010 [21]	Acupuncture plus cupping therapy and amitriptyline versus amitriptyline	-4.70 [−5.67, −3.73]		
Overall (fixed model)		**MD −4.59** [**−5.49, −3.69**]	**<0.00001**	**0%**
(3.3) Therapeutic effect of combination of acupuncture and point injection				
Liu 2012 [24]	Acupuncture combined with point injection (Vit B12) versus amitriptyline	MD −1.50 [−3.46, 0.46]	0.13	NA

(4) Assessment for depression after treatments				
(4.1) Therapeutic effect of acupuncture				
(4.1.1) Electroacupuncture versus sham electroacupuncture (FIQ)				
Martin et al. 2006 [25]	Electroacupuncture versus sham electroacupuncture without insertion	SMD −0.33 [−0.90, 0.23]	0.25	NA
(4.1.2) acupuncture versus anti-depression drugs				
Gong and Wang 2010 [17]	Acupuncture versus amitriptyline (HAMD)	−0.78 [−1.30, −0.25]		
Hadianfard and Parizi 2012 [18]	Acupuncture versus fluoxetine (FIQ)	−0.48 [−1.20, 0.25]		
Overall (fixed model)		**SMD −0.67** [**−1.10, −0.25**]	**0.02**	**0%**
(4.2) Therapeutic effect of combination of acupuncture and cupping therapy (HAMD)				
(4.2.1) Combination of acupuncture and cupping therapy versus western medications				
Jiang et al. 2010 [21]	Acupuncture plus cupping therapy and amitriptyline versus amitriptyline	MD 0.90 [−0.68, 2.48]	0.26	NA
(4.2.2) combination of acupuncture and cupping therapy plus medications versus medications alone				
Cao and Li 2003 [15]	Acupuncture plus cupping therapy and seroxat versus seroxat alone	MD −6.00 [−8.36, −3.64]	<0.00001	NA
Jiang et al. 2010 [21]	Acupuncture plus cupping therapy and amitriptyline versus amitriptyline	MD −1.78 [−2.97, −0.59]	0.003	NA

(5) Assessment for sleep quality after treatments				
(5.1) therapeutic effect of acupuncture				
Gong and Wang 2010 [17]	Acupuncture versus amitriptyline	−0.34 [−0.85, 0.17]		
Liu 2012 [24]	Acupuncture versus amitriptyline	−0.11 [−0.74, 0.52]		
Yao 2006 [29]	Acupuncture versus amitriptyline	−0.43 [−0.93, 0.07]		
Overall (fixed model)		**SMD −0.32** [**−0.63, −0.01**]	**0.04**	**0%**
(5.2) Therapeutic effect of combination of acupuncture and point injection				
Liu 2012 [24]	Acupuncture combined with point injection (Vit B12) versus amitriptyline	SMD −0.94 [−1.46, −0.42]	0.0004	NA

(6) FQI after treatments				
(6.1) Therapeutic effect of acupuncture				
(6.1.1) Electroacupuncture versus sham electroacupuncture				
Martin et al. 2006 [25]	Electroacupuncture versus sham electroacupuncture without insertion	MD −4.30 [−11.08, 2.48]	0.21	NA
(6.1.2) Acupuncture versus antidepression drugs				
Hadianfard and Parizi 2012 [18]	Acupuncture versus fluoxetine	MD −4.60 [−12.42, 3.22]	0.25	NA

(7) Assessment for fatigue after treatments				
(7.1) Therapeutic effect of acupuncture				
(7.1.1) Acupuncture versus sham acupuncture				
(7.1.1.1) Electroacupuncture versus sham electroacupuncture				
Martin et al. 2006 [25]	Electroacupuncture versus sham electroacupuncture without insertion	SMD −0.23 [−0.79, 0.33]	0.42	NA
(7.1.1.2) acupuncture versus sham acupuncture				
Harris et al. 2005 [19]	Acupuncture on traditional site versus acupuncture on nontraditional site	0.11[−0.50, 0.71]		
Harris et al. 2005 [19]	Acupuncture on traditional site with stimulation versus acupuncture on nontraditional site with stimulation	0.01 [−0.67, 0.68]		
Subtotal (fixed model)		SMD 0.06 [−0.39, 0.51]	0.79	0%
Overall (fixed model)		**SMD −0.05** [**−0.41, 0.30**]	**0.77**	**0%**
(7.1.2) Acupuncture versus antidepression drugs				
Hadianfard and Parizi 2012 [18]	Acupuncture versus fluoxetine	SMD -0.27 [−0.99, 0.45]	0.46	NA

MD: mean difference.

TCM: traditional Chinese medicine.

TENS: transcutaneous electrical nerve stimulation.

RR: risk ratio.

**Table 3:** Characteristic of randomized controlled trials outside meta-analysis.

Study ID	Comparisons	Main findings
Acupuncture versus sham acupuncture		
Lautenschlager 1989 [22]	Acupuncture versus sham laser acupuncture	There was significant difference between acupuncture and sham treatment in pain reduction measured for all 3 methods by end of treatment. At follow up of 3 months after the last treatment, no significant changes were observed (*P* > 0.05).

Sprott 1998 [27]	Acupuncture versus sham laser acupuncture	The data for pain reduction by tender points were not completely reported, but the results showed that the number of tender points was not significantly decreased after acupuncture treatment in comparison to sham treatment (*P* > 0.05). The intensity of pain, measured by the VAS, also showed no significant reduction, neither immediately at the end of treatment or 2 months after the treatment (*P* > 0.05).

Acupuncture versus no treatment		
Sprott 1998 [27]	Acupuncture versus no treatment	The number of tender points was significantly decreased after acupuncture treatment in comparison to no treatment (*P* > 0.05).

Acupuncture plus standard cares versus standard cares alone		
Targino 2008 [28]	Acupuncture plus tricyclic antidepressants and exercise with tricyclic antidepressants and exercise	Patients in acupuncture group were significantly better than the control group in terms of VAS scores (*P* < 0.001), pressure pain threshold (PPT) (*P* < 0.001), the number of tender points below 4 kg/cm^2 ^(*P* < 0.001), and in 5 subscales of the SF-36 (*P* < 0.05).
